# Holding services to account

**DOI:** 10.1111/j.1365-2788.2008.01068.x

**Published:** 2008-07

**Authors:** J Clegg

**Affiliations:** School of Community Health Sciences, University HospitalNottingham, UK

**Keywords:** audit, evaluation, Healthcare Commission, value for money

## Abstract

**Background:**

Recently, the frequency of audit inspections of health services for people with intellectual disability (ID) in the UK has increased, from occasional inquiries to a systematic audit of all services. From 2008, a process of continuous audit ‘surveillance’ of specialist health services is to be introduced. Similar regimes of inspection are in place for social care services.

**Aim:**

To explore the conceptual positions which inform audit, through detailed examination of the investigation into the learning disability service at Sutton and Merton.

**Findings:**

Audit is distinct from evaluation because it neither provides opportunities for service staff to give an account of their work nor represents a search for knowledge. Audit investigates adherence to government policy. In ID, audits measure aspirations derived from normalisation, despite research showing that some of these aspirations have not been achieved by any service. As audit consumes significant public resource, it is questionable whether the dominant finding of the Healthcare Commission's investigation into Sutton and Merton, that the ID service was chronically under-funded, represents value for money.

**Discussion and conclusions:**

While basic checks on minimum standards will always be necessary, service excellence requires not audit but research-driven evaluation. Audits inhibit rather than open-up debate about improving support to people with ID. They impose an ideology, squander resource, and demoralise carers and staff. Evaluations challenge the implicit management-versus-professional binary enacted by audit, and can inform new care systems which make effective use of all those engaged with people with ID.

## Introduction

History has repeatedly shown that people with intellectual disability (ID) are vulnerable to abuse of all types, from all quarters: ‘*Both* institutions and communities can be inhumane and exploitative; each mirrors the world around us’. (Trent 1994, p. 277, his italics). Improving the lives of people with ID by enhancing the quality of services is a concern shared by professionals, staff and carers. It is also demanded by history, which shows that services for people with ID who set up with minimally adequate funds tend to be pared back until they reach unacceptable levels. For example, Thomson (1998) described the first stage of community care for people with ID in late 1950s, Britain, as follows:

‘There was a belief that problems could be solved by administrative reorganization. … Questions of resources and finance, vital to any effective caring policy, were either evaded or relied unrealistically upon reallocation of patients from hospital to community care … The level of expenditure per head … had probably fallen over the 1950s … shamefully, government expenditure in 1960 was less than that spent on compensation for fowl pest’. (pp. 294–295)

The Ely Hospital Inquiry (Department of Health & Social Security 1969) prompted the allocation of significant funds to the All Wales Strategy, a community development project. The All Wales Strategy's aspiration was that services would be based on individual plans, but this was never achieved: at best it covered one-third of the ID population and could be as low as 10% (Felce 2004). Felce also criticised planning based solely on the identification of individual need, not only because it has never been close to being achieved, but also because indicative targets are necessary to ensure adequate levels of service provision. A 12-year follow-up of a hospital closure project (Hallam *et al*. 2006) during a period of increased service individualisation evidences the weakness of service planning based on identification of individual need. They found that increased funds made available to move hospital residents into the community steadily declined: adjusted to 2002–2003 rates, weekly mean expenditure per person was £736 before hospital closure, £899 1 year later, £871 5 years later and only £765 at the 12-year follow-up. Many apparent indicators of the benefits of community care 1 year after hospital closure decreased commensurately with funding reductions across those 12 years.

Services for people with ID in England have recently become the subject of public concern again, this time, attracting inquiries by a national body, the Commission for Healthcare Audit and Inspection (shortened to Healthcare Commission). Following investigation into complaints about two services in 2006, the Healthcare Commission decided to audit all specialist inpatient healthcare services for people with ID across England during 2007.

## Aims of this analysis

This paper examines the conceptual basis of recent audits of English ID services and their effects, through detailed analysis of the *Investigation into the service for people with learning disabilities provided by Sutton and Merton Primary Care Trust* (Healthcare Commission 2007a) (the Investigation). Power's (1997) deconstruction of the conceptual basis of UK public service audits informs this analysis of the aims, epistemology, recommendations and effects of that Investigation.^1^ It ends by drawing recursively on a key audit criterion to consider the Investigation's value for money.

## Purpose of the Sutton and Merton investigation

Sutton and Merton Primary Care Trust (PCTs are responsible for commissioning health services in each district of the UK) invited the Healthcare Commission to investigate its ID service after a number of serious incidents, including allegations of physical and sexual assault. The Investigation aimed ‘to establish whether the ways of working at the PCT were adequate to ensure both the safety of the people using the service and the quality of the service provided’. (Healthcare Commission 2007a). The specialist health service for people with ID in the 95-bedded Orchard Hill hospital was to be audited alongside services for people living in National Health Service (NHS) community homes. Relevant context was that two attempts to close Orchard Hill had been subject to legal challenge: a 2001 judicial review of the decision-making process, and applications to the High Court in 2004 that hospital closure was not in the best interests of two people. These were withdrawn in 2005 following an agreement brokered by the official solicitor that 38 people with ID would have their needs assessed by independent experts, but another proposed closure date had to be further delayed to 2009 because of problems recruiting these experts.

The PCT appears to have been caught between a government policy which required service closure and a parents’ association with sufficient legal acumen to resist it. Uncertainty about a service's future generally encourages staff to seek stable employment elsewhere; it makes ensuing vacancies look unattractive, and so forces increased use of agency staff; it also discourages investment in building maintenance. These factors feed service demoralisation and poor practice. Combined with frequent reorganisations which distracted the purchasing authorities responsible for the service, the scene was set for serious incidents in a service which was proving difficult to manage. It is possible that an unspoken aim of the investigation was to strengthen the PCT's arm against a parents’ association which was challenging government policy.

## Epistemology

Epistemology refers to the theory of knowledge and the relationship between beliefs and knowledge, which inform practice. The ‘philosopher-accountant’Power (1997) provides a historical and contemporary analysis of the epistemology of audit. In 1866, Gladstone established the role of ‘Comptroller and Auditor General’ to evaluate only the means, not the ends, of government programmes. Power tracks this through the development of financial audits on which public service audits are based. Financial audits make no statement about a company's status or future profitability; they merely sample documents and processes, in order to generate an opinion about whether or not accounts presented to Annual General Meetings of shareholders represent a ‘true and fair account’ of that company's financial dealings.

Power asserts that although they may be mistaken for evaluations, audits are not evaluations as social scientists understand them: audit is an expression of New Public Management where states use quasi-markets to exert indirect but nevertheless close control over services. He argues that audits are used across the developed world to harness spiralling public spending, but that neo-liberal governments have elevated the normative check which audits provide. While evaluations examine causes, effects and their relationship, he argues that audits are a management tool to silence professionals. ‘NPM claims to speak on behalf of taxpayers and consumers and against cosy cultures of professional self-regulation’. (Power 1997, p. 44). Despite the arbitrariness of measures employed by auditors, their replicability and consistency are preferred to the nuances, ambiguities and qualifications which evaluators and researchers generate. Power states that audit imposes downward accountability: none of those audited are allowed to offer their own account of what is happening or has happened.

Examination of the measures used in an audit reveals its conceptual underpinnings. The Investigation follows a tradition in ID established by the first normalisation assessment tool, Program Analysis of Service Systems (Wolfensberger & Glenn, 1972), of assessing services against aspirations rather than against criteria known to be achievable. The main measures of quality of care in the Investigation were 10 ‘indicators’ drawn up by British Institute of Learning Disability (BILD). There indicators were apparently the outcome of a process of consultation with people with ID in 21st century Britain, but they bear a striking resemblance to O'Brien's (1987) North American ‘five accomplishments’ (Choice, Participation, Community Presence, Competence, Respect). BILD's term ‘indicators’ implies they have evidential support, but they are simply culturally available narratives derived from the normalisation principle. Their familiarity makes them difficult to dispute: ‘Surely you are not suggesting people with ID should be treated with *dis*respect!’, but using such aspirations as audit measures presents at least three types of problem.

First, the ‘indicators’ are not accompanied by consideration of when they should or should not apply. For example, while people may want to make decisions about everyday and significant moments in their lives, people may also encounter times when they want others to decide for them or at least advise them. The latter is more likely when people are ill, confused or lack crucial knowledge: integral experiences for most people with severe ID.

Second, some of the ‘indicators’ of quality ignore evidence that even good services rarely exhibit them. For example, indicators requiring community integration and a range of social relationships ignore research showing that these are difficult attainments for people with ID with few additional problems (Cummins & Lau 2003; Forrester-Jones *et al*. 2006), while many of those using the Sutton and Merton service were profoundly disabled and had additional complex needs. Similarly, obtaining work and/or meaningful occupation is difficult for adults with severe ID (Clegg *et al*. 2008).

Finally, the Investigation was critical of the failure to ensure that each person had an individual assessment of their needs; yet this policy aspiration has not been achieved across any service. Reflecting on the All Wales Strategy which first attempted to introduce individualised planning, Felce made the following comment: ‘Anyone unconvinced by the enormity of the development agenda should explore the lessons from the low rate of implementation of individual planning in Wales. … The sad fact was that there was never the capacity necessary to establish regular individual plans for people with ID in Wales, despite the largest central government investment aimed towards people with ID in Britain in recent times’. (Felce 2004, p. 28 n. 1).

## Recommendations and effects

Judging from the way Healthcare Commission reports its work, the number of ‘recommendations for improvement’ made is considered to reflect the competence or otherwise of services. Despite acknowledging that their recommendations range from ‘fairly minor suggestions’ to ‘major breaches of a serious nature’, basic service evaluation methodology is transgressed by simple addition of such different types of data.

The Investigation recommended that services should have enough qualified nurses and care staff to enable severely disabled people with complex needs to go out and be active, maintain appropriate records, to ensure that staff are managed effectively, can attend mandatory training and digest burgeoning numbers of policies; they should also employ enough psychologists, psychiatrists and speech therapists to allow the service to provide best-practice therapies, training, clinical supervision and care-planning. As these time-consuming activities are resource-intensive, more than half of these recommendations respond to one problem: under-funding.

Another recommendation required the development of an easy-read version of the complaints procedure. Power's general warning about audit is pertinent here: ‘Practices are perpetuated isomorphically because they have become legitimate and not necessarily because they have been even moderately effective in achieving goals’. (Power 1997, p. 145). The frequently repeated policy requirement that services should provide ‘accessible’ information was recently questioned by Poncelas & Murphy (2007) who showed that adding symbols to simple texts does not improve understanding because understanding depends upon language comprehension. Half of the severely disabled hospital population in Sutton and Merton were non-ambulant and had difficulties eating or swallowing: their language comprehension will have been extremely limited. Yet such considerations appear to have been ignored in the Investigation's requirement that ‘easy-read’ information should be available for residents’‘empowerment’.

Did the Investigation meet its stated aim, to investigate whether or not the PCT's ways of working were adequate to ensure the safety of the people using the service and service quality? The PCT announced to the press (The Independent 22.2.07), in advance of the investigation report being published, that during the previous year they had increased the number of staff employed in the service by 50%, overhauled management, and provided better training. It, therefore, appears that there was no need for an inquiry: the PCT already knew that many problems were caused by chronic under-resourcing. The Investigation stated that the PCT would be unlikely to be able to close Orchard Hill and implement new ways of working without ‘adequate transitional financial arrangements’ (Healthcare Commission 2007a, p. 8). As the PCT could suddenly afford to increase its staff, presumably additional funds were made available. This suggests that the Investigation's main effect was not scrutiny of the PCT's ways of working but enabling Sutton and Merton PCT to access previously unavailable funds.

There is an important shift in perspective between the full report and shortened versions. The full report did explore and criticise the PCT's ways of working. It noted interaction between ID being a small part of the PCT's portfolio of services and four major reorganisations involving seven chief executives, which resulted in nobody taking responsibility for the poor quality of the ID service. Yet managerial failure is minimised in the brief summary of the Investigation: ‘Our investigation report details how outmoded, institutionalised care led to the neglect of people with learning disabilities at Sutton and Merton Primary Care Trust’. (Healthcare Commission 2007b), an assertion repeated in the press statement to accompany the publication of the Investigation (The Independent 22.2.07). As Gleeson & Kearns (2001) observed, responsibility shifts very easily onto the shoulders of the least powerful: the care staff. No wonder they become demoralised.

Was it an unspoken aim of the investigation to strengthen the PCT against some troublesome parents? Despite concern to ensure that they had information about the complaints procedure, the parents’ reasons for resisting hospital closure were neither explored nor addressed. Two recommendations merely confirm the closure plans. In January 2008, the ex-secretary of the registered charity Orchard Hill Parents and Staff Association confirmed that the Association is no longer in existence. Effectively, the troublesome parents were vanquished by the process.

## Value for money

‘Methods of checking and verification are diverse, sometimes perverse, sometimes burdensome and always costly’. (Power 1997, p. 1). The total Healthcare Commission budget (Healthcare Commission 2007c) is stated to be £69.8 billion. If this astonishing figure from their corporate report is accurate, according to Public Expenditure Statistical Analysis (2008) of the most recent data available (2005–2006) the Healthcare Commission budget represents twice the total amount spent on defence (£30.7 billion) and equals the total education budget (£69.3 billion).

How much did this particular Investigation cost? Last year, £11 800 000 was spent on ‘Safeguarding the public’ (Healthcare Commission 2007c): handling about 8000 second stage NHS complaints, and investigating four serious service failures, of which Sutton and Merton was one. If total complaints-handling is equivalent to one formal investigation of service failure, then this investigation probably cost around £2 400 000.

So the evidence appears to confirm Power's claim that audit is costly. It might still be value for money, although Power doubts it: ‘Audit is a practice which in every sphere where it operates must necessarily talk up expectations … The ‘expectations gap’ is not so much a problem for auditing as its constitutive principle’. (Power 1997, p. 144). The following claims made in its Corporate Plan might indeed be regarded as talking up expectations: ‘We provide authoritative information about the quality of healthcare services and we make sure that this is useful to a variety of audiences’. (Healthcare Commission 2007c, p. 3) and ‘We will provide more and better comparative information on services that will be useful to providers, commissioners and the public’. (p. 12).

Yet as we have seen, the PCT already knew that the service was significantly under-resourced. It is doubtful that the government needed to spend around £2.4 million to find out that ‘Historically, staffing levels were low, with a reliance on temporary NHS and agency staff’ (Healthcare Commission 2007a, p. 5) and ‘The number of staff was insufficient to meet the needs of the service’ (Healthcare Commission 2007a, p. 55).

## Discussion

It seems that the pattern observed by historians, of official neglect of ID services followed by critical inquiries which release resource, continues. Although inspection is usually uncomfortable for the staff examined, increased service scrutiny is generally accepted as an improvement on the more common experience, of senior managers considering ID unworthy of any attention at all. Yet audit is not evaluation, and lessons from an audit of another English service question the wisdom of such acceptance.

Stronach (2006) summarised the outcome of recent events in the life of Summerhill, an independent school established in 1921 to allow children to grow emotionally by removing fear and coercion by adults. A key principle is that children do not have to attend lessons. In 1999, an Office for Standards in Education (OFSTED) inspection issued Summerhill with a Notice of Complaint which required it to ensure that children did attend lessons. The school launched an appeal which was heard by a High Court Tribunal in 2000: evidence for it was provided by an independent academic evaluation of the school funded by the Nuffield Institute and carried out by Stronach. After only 3 days of the hearing, counsel for the Department for Education and Employment withdrew its notice of complaint. A subsequent OFSTED inspection of Summerhill, reported in *The Guardian* newspaper (1.12.07), found pupils’ social and personal development to be ‘outstanding’; the head teacher emphasised that it was not the school that had changed but the inspectorate, which now vindicated their philosophy. Stronach (2006) notes that while OFSTED emphasise that their inspections are not based on any particular school of theory, their Framework requirements include unproven axioms concerning ‘minimum standards’ and ‘efficient and suitable’ education. Parents likened OFSTED's 1999 audit of Summerhill to a game of tennis judged by the rules of basketball.

The Summerhill story demonstrates Power's key distinction between audit and evaluation: audit represents ideological imposition; evaluation provides a nuanced account based on evidence and argument. ‘Most audit reports function as labels which must be trusted. They do not form the basis for communication and dialogue’. (Power 1997, pp. 127–128). Yet accounts and account-giving are part of what it is to be a rational individual (Douglas 1992). Audits are communicated with excessive certainty and in a manner which deters curiosity, a style which fits particularly well with ID culture criticised by Gleeson & Kearns (2001) for ‘excessive certitude’. Together, they generate such high levels of certainty that critiques appear unable to effect policy. Neither Felce's (2004) criticism of individualised planning nor the germ of parental opposition in Sutton and Merton has been able to generate the successful policy challenge raised by Summerhill.

The Department of Health, through the Healthcare Commission, has declared a process of continuous audit of all ID services. Ten years ago, Power argued that Britain was becoming an audit society which ‘invests too heavily in shallow rituals of verification at the expense of other forms of organisational intelligence’. (Power 1997 p. 123) He warned that societies which have institutionalised checking on a grand scale have crumbled: staff become weighed down by demands for information, while resource needed to support people with ID is squandered on surveillance. The move from occasional to continuous audit based on the same criteria suggests that Power's predictions are coming to pass.

## Conclusions

The Healthcare Commission's investigation of Sutton and Merton emerged out of, and entrenched, inappropriate levels of certainty about good practice in ID. It used questionable measures: some service aspirations have come to positive fruition, but others have come no closer despite more than a quarter of century of effort. It cannot be considered to be value for money. We need clear statements about how much money should be allocated to provide a minimum level of service for people with ID, and brief inspections which check that minimum levels of provision are maintained. But more important would be a return to service evaluations which draw on research methodology and encourage debate. Evaluation should involve all stakeholders: no good purpose is served by reinforcing the management-versus-professional moral binary supported by audit, nor by vanquishing parental opposition. Less resource should be devoted to ritually checking the mundane, in order to release the time, energy and money to explore new and different ways to improve the lives of people with ID.

## Footnotes

1 Professor Power is employed by the London School of Economics and Political Science, a UK university, where he is head of the Centre for Analysis of Risk and Regulation, funded by the Economic and Social Research Council. Power continues to draw on his 1997 analysis of the ‘Audit Society’, e.g. in 2003: ‘Auditing and the production of legitimacy’, *Accounting, Organizations and Society v28*, 379–94. Other researchers also draw on Power to inform analysis of different non-financial audits, for example this Canadian research team: Free, C. Salterio, S. E. & Shearer, T. (in press). The construction of auditability: MBA rankings and assurance in practice. *Accounting, Organizations and Society* available online from 20/3/08.
